# Evaluation of Antioxidant and Immunity Activities of Quercetin in Isoproterenol-Treated Rats

**DOI:** 10.3390/molecules17044281

**Published:** 2012-04-10

**Authors:** Hui Liu, Lei Zhang, Shaoping Lu

**Affiliations:** 1 Department of Cardiology, Tangdu Hospital, Fourth Military Medical University, Xi’an 710038, Shaanxi, China; 2 Department of Internal Medicine, The People’s Hospital of Long Xian, Long Xian 721200, Shaanxi, China

**Keywords:** isoproterenol, quercetin, ischemia, myocardial, antioxidant, immunity

## Abstract

The present study was designed to evaluate the effect of quercetin on myocardial oxidative stress and immunity function impairment induced by isoproterenol in rats. To induce myocardial ischemia, Wistar rats were subcutaneously injected with isoproterenol (70 mg/kg). Blood immunity index, cardiac marker enzymes and antioxidative parameters in hearts were measured. It was found that the levels of blood AST, creatine kinase, NO, NOS, IL-10, IL-1, IL-8 and lactate dehydrogenase in isoproterenol-treated rats were significantly increased. The rats administrated with isoproterenol showed the declines in myocardial antioxidant enzymes activities. Administration of quercetin significantly ameliorated myocardial oxidative injury and immunity function impairment induced by isoproterenol. The results indicated that quercetin possesses activity against isoproterenol-induced myocardial oxidative injury and immunity function impairment, and that the mechanism of pharmacological action was related at least in part to the antioxidant activity of quercetin.

## 1. Introduction

Lack of blood supply or ischemia underlies many of the most important diseases faced by clinicians in their daily practice, including myocardial infarction, angina pectoris, thrombotic stroke, *etc*. [1]. The treatment of this condition has extended a new era where mortality can be approximately halved by procedures which allow the rapid return of blood flow, *i.e.*, reperfusion, to the ischemic zone of myocardium [2]. Myocardial ischemia and reperfusion can generate oxygen-derived free radicals that are harmful to the ischemic heart tissue [3,4,5,6]. Oxygen radicals may react with tissue components to form metabolites that can be used to evaluate the status of oxidative stress.

Experimental models using aortic or renal artery constriction, which induce cardiac hypertrophy by pressure-overload, and exercise-training, which induces cardiac hypertrophy by volume overload, demonstrated an increased susceptibility to ischemia compared to normal myocardium [7,8]. Another, non-surgical technique to induce cardiac hypertrophy in rats uses subcutaneous injections of the catecholamine isoproterenol (ISO). ISO is used in this study to evaluate the protective effect of various drugs on cardiac function. It causes severe stress in the myocardial tissue and its high doses produce acute myocardial necrosis.

Quercetin is one of the most representative compounds in the flavonoid family and has been attributed to a number of potential health benefits, including antioxidant, cancer prevention, DNA protection, anti-inflammatory action, and cardioprotective activity [9,10,11,12,13,14,15,16,17]. The oral administration of quercetin increased plasma antioxidant capacity and protected against hepatic ischemia–reperfusion injury significantly in rats [18,19,20,21]. Additionally, quercetin has protective effects on cell function *in vitro* and *in vivo* [22,23].

## 2. Result

Compared with normal control, electrocardiogram T wave height in ISO-treated rats was significantly decreased (*p* < 0.01), whereas heart index was markedly increased (*p* < 0.01). Compared with ISO-treated rats, three doses of quercetin treatment significantly enhanced T wave height and decreased heart index (*p* < 0.05, *p* < 0.01). The effect was dose-dependent (Table 1).

**Table 1:** Effect of quercetin treatment on T wave height and heart index.

Group	T wave height	Heart index
Normal control	2.68 ± 0.24	2.85 ± 0.19
ISO control	0.93 ± 0.08 ^b^	3.23 ± 0.28 ^b^
ISO + quercetin (50 mg/kg b.w.)	1.48 ± 0.13 ^d^	3.2 ± 0.33
ISO + quercetin (100 mg/kg b.w.)	1.99 ± 0.2 ^d^	3.17 ± 0.29
ISO + quercetin (150 mg/kg b.w.)	2.36 ± 0.27 ^d^	2.97 ± 0.26 ^c^

^b^ *P* < 0.01, compared with normal control group; ^c^ *P* < 0.01, ^d^*P* < 0.01, compared with ISO control group.

In this study, significant difference in initial body weight of rats wasn’t found to be significant between groups. Body weight gain was not significantly different between all the groups. However, weight gain was slightly lower in ISO rats as compared to normal control group.

Table 2 shows the effect of quercetin on plasma AST, CKMB, LDH and TNF-α activities in IR rats. There was a significant increase (*p* < 0.01) in plasma AST, CKMB, LDH and TNF-α activities in ISO control rats as compared to normal control group. There was a significant decrease (*p* < 0.01) in plasma AST, CKMB, LDH and TNF-α activities in group ISO + quercetin (50, 100 and 150 mg/kg b.w.) as compared to ISO control. The effect was displayed in a dose-dependent manner.

**Table 2:** Effect of quercetin on plasma AST, CKMB, LDH and TNF-α activities in ISO rats.

Group	AST (U/L)	CKMB (U/L)	LDH (U/L)	TNF-α (ng/mL)
Normal control	231.81 ± 17.04	3,418.32 ± 276.39	1,265.17 ± 104.21	1.68 ± 0.09
ISO control	383.08 ± 21.62 ^b^	7,217.39 ± 401.62 ^b^	2,971.82 ± 174.24 ^b^	3.07 ± 0.23 ^b^
ISO + quercetin (50 mg/kg b.w.)	341.35 ± 19.48 ^d^	5,935.14 ± 276.13 ^d^	2,518.42 ± 128.41 ^d^	2.64 ± 0.17 ^d^
ISO + quercetin (100 mg/kg b.w.)	295.14 ± 13.07 ^d^	4,705.18 ± 201.52 ^d^	2,105.37 ± 153.11 ^d^	2.06 ± 0.11 ^d^
ISO + quercetin (150 mg/kg b.w.)	249.11 ± 14.16 ^d^	4,061.64 ± 222.19 ^d^	1,682.16 ± 125.83 ^d^	1.89 ± 0.16 ^d^

^b^ *P* < 0.01, compared with normal control group; ^d^ *P* < 0.01, compared with ISO control group.

Table 3 shows the effect of quercetin on plasma NO, NOS, IL-1, IL-8 and IL-10 levels in ISO rats. There was a significant decrease (*p* < 0.01) in plasma NO, NOS and increase in plasma IL-1, IL-8 and IL-10 levels in ISO control rats as compared to normal control group. There was a significant increase (*p* < 0.01) in plasma NO, NOS, IL-10 and decrease in IL-1, IL-8 levels in group ISO + quercetin (50, 100 and 150 mg/kg b.w.) as compared to ISO control group. The effect was displayed in a dose-dependent manner.

**Table 3:** Effect of quercetin on plasma NO, NOS, IL-1, IL-8 and IL-10 levels in ISO rats.

Group	NO (μmol/L)	NOS (U/mL)	IL-1 (ng/L)	IL-8 (ng/L)	IL-10 (ng/L)
Normal control	37.14 ± 1.21	17.03 ± 1.03	36.43 ± 1.11	97.28 ± 4.02	1.73 ± 0.09
ISO control	14.82 ± 0.95 ^b^	9.05 ± 0.73 ^b^	147.21 ± 12.08 ^b^	191.06 ± 11.82 ^b^	33.18 ± 1.03 ^b^
ISO + quercetin (50 mg/kg b.w.)	19.47 ± 0.94 ^d^	13.76 ± 0.49 ^d^	112.15 ± 7.04 ^d^	162.11 ± 9.04 ^d^	39.12 ± 1.38 ^c^
ISO + quercetin (100 mg/kg b.w.)	25.17 ± 1.33 ^d^	15.13 ± 0.73 ^d^	89.53 ± 3.28 ^d^	138.35 ± 8.93 ^d^	42.61 ± 2.63 ^d^
ISO + quercetin (150 mg/kg b.w.)	34.01 ± 1.29 ^d^	16.94 ± 0.82 ^d^	66.37 ± 3.17 ^d^	106.3 ± 5.38 ^d^	47.09 ± 2.05 ^d^

^b^ *P* < 0.01, compared with normal control group; ^c^ *P* < 0.05; ^d^ *P* < 0.01, compared with ISO control group.

The activities of Na^+^-K^+^-ATPase, Ca^2+^-Mg^2+^-ATPase and MPO in the heart of IR rats were described in Table 4. In IR rats, mycardium Na^+^-K^+^-ATPase, Ca^2+^-Mg^2+^-ATPase and MPO activities in ISO control group were significantly (*p* < 0.05) decreased, whereas MPO activity was significantly increased compared to normal control group. Treatment of ISO rats with quercetin (50, 100 and 150 mg/kg b.w.) dose-dependently significantly enhanced myocardium Na^+^-K^+^-ATPase, Ca^2+^-Mg^2+^-ATPase activities and decreased the MPO activity compared to ISO control group (*p* < 0.01).

**Table 4:** Effect of quercetin on mycardium Na^+^-K^+^-ATPase, Ca^2+^-Mg^2+^-ATPase and MPO activities in ISO rats.

Group	Na^+^-K^+^-ATPase (μmol Pi/mg prot/h)	Ca^2+^-Mg^2+^-ATPase (μmol Pi/mg prot/h)	MPO (U/g)
Normal control	5.05 ± 0.14	6.14 ± 0.42	4.06 ± 0.25
ISO control	3.29 ± 0.15 ^b^	3.97 ± 0.13 ^b^	19.06 ± 0.94 ^b^
ISO + quercetin (50 mg/kg b.w.)	4.42 ± 0.16 ^d^	4.44 ± 0.26 ^c^	14.32 ± 0.82 ^d^
ISO + quercetin (100 mg/kg b.w.)	4.81 ± 0.22 ^d^	5.39 ± 0.21 ^d^	10.71 ± 0.82 ^d^
ISO + quercetin (150 mg/kg b.w.)	4.92 ± 0.24 ^d^	5.84 ± 0.37 ^d^	7.03 ± 0.46 ^d^

^b^ *P* < 0.01, compared with normal control group; ^c^ *P* < 0.05; ^d^ *P* < 0.01, compared with ISO control group.

There was significant (*p* < 0.05) increase in myocardial TBARS level and decrease in myocardial GSH level, SOD, CAT, GSH-Px activities in the ISO control group when compared to the normal control (Table 5). Significant decrease in the level of myocardial TBARS and increase in the level of myocardial GSH and the activities of SOD, CAT and GSH-Px were observed in groups ISO + quercetin (50, 100 and 150 mg/kg b.w.) (*p* < 0.01) in comparison to the ISO control group. Moreover, the effect was displayed in a dose-dependent manner.

**Table 5:** Effect of quercetin on myocardium TBARS, GSH levels and SOD, CAT, GSH-Px activities in ISO rats.

Group	TBARS (nmol/g of tissue)	GSH (nmol/mg protein)	SOD (U/mg protein)	CAT (U/mg protein)	GSH-Px (U/mg protein)
Normal control	4.17 ± 0.31	69.25 ± 2.99	267.28 ± 11.73	49.14 ± 1.54	68.14 ± 4.02
ISO control	8.41 ± 0.47 ^b^	32.16 ± 1.42 ^b^	98.27 ± 4.29 ^b^	18.23 ± 0.95 ^b^	32.07 ± 1.32 ^b^
ISO + quercetin (50 mg/kg b.w.)	6.94 ± 0.23 ^d^	41.07 ± 2.07 ^d^	153.28 ±14.02 ^d^	26.07 ± 1.44 ^d^	45.18 ± 2.05 ^d^
ISO + quercetin (100 mg/kg b.w.)	6.03 ± 0.29 ^d^	53.24 ± 3.01 ^d^	197.04 ± 12.16 ^d^	35.19 ± 1.53 ^d^	57.02 ± 2.51 ^d^
ISO + quercetin (150 mg/kg b.w.)	5.26 ± 0.19 ^d^	66.93 ± 2.18 ^d^	241.71 ± 18.02 ^d^	48.01 ± 2.97 ^d^	66.32 ± 3.13 ^d^

^b^ *P* < 0.01, compared with normal control group; ^d^ *P* < 0.01, compared with ISO control group.

On histopathological examination, large areas of coagulative necrosis were seen in isoproterenol-treated rats, with neutrophilic infiltrate, diffused interstitial edema and pale myocytes with fading nuclei and decreased striations. Pathological features of the infarct area became apparent with widespread necrosis, including the presence of contraction bands, polymorph nuclear leukocytes infiltration, capillaries compressing and a lot of hemorrhage. After three doses of quercetin treatment, the histological features became typical of normal cardiac structure or mild architectural damage, characterized by interstitial edema and localized necrotic areas.

## 3. Discussion

Isoproterenol-induced myocardial ischemia has been reported to show many metabolic and morphologic aberrations in experimental animals similar to those in human. Isoproterenol causes subendocardial myocardial ischemia, hypoxia, necrosis, which results in the decrease of myocardial compliance and inhibition of diastolic and systolic function. Therefore isoproterenol is widely used as an agent to evaluate the effect of drugs in the myocardial consequences of ischemic disorders [24].

Myocardial cell contains marker enzymes such as AST, CKMB and LDH. Myocardial ischemia induces cell membrane to permeate or rupture, which results in the leakage of the CKMB, AST and LDH into blood. Hence, the CKMB, AST and LDH activities in serum reflect the alterations of membrane integrity and the degree of myocardial injury [25]. Our results showed that isoproterenol injection caused a significant elevation in AST, CKMB and LDH activities. However, administration with quercetin at a dose of 50, 100 or 150 mg/kg significantly lowered the isoproterenol-induced increase in AST, CKMB and LDH activities. The results demonstrated that quercetin could attenuate myocardial ischemia injury induced by isoproterenol.

Nitric oxide (NO) is an important regulator of endothelial functions and vascular tone. It is an inhibitor of platelet aggregation in biological tissues and may also exert a significant influence in several pathological conditions [26,27,28]. NO is synthesized from L-arginine in the presence of O_2_ and NADPH-diaphorase in a reaction catalyzed by nitric oxide synthase (NOS). NOS is a family of enzymes with three isoforms, neuronal NOS (nNOS), endothelial NOS (eNOS) and inducible NOS (iNOS) [29]. Recent data show that NOS activity could be modified by ischemia, correlating blood flow oxygen supply and damage. Many cases report that the chronic exposure to hypoxia and hyperoxia may induce a variation of eNOS protein in many tissues [30].

Reperfusion of the ischemic myocardium is associated with a dramatic inflammatory response leading to TNF-alpha (TNF-α) release, interleukin-10 (IL-10) induction, and subsequent neutrophil-mediated cytotoxic injury [31]. The anti-inflammatory and anti-atherogenic properties of IL-10 have been demonstrated using several models of atherosclerosis in mice [32]. In humans, the expression of IL-10 has been demonstrated in both coronary arteries and atherosclerotic plaque [33]. IL-10 inhibits synthesis of various cytokines (including IL-1, TNF-α, IL-6 and IL-8) by stimulated monocyte/macrophages, suppressing the inflammatory response [34,35,36,37]. Recently, much interest has been focused on the potential role that IL-10 plays a pivotal role in the myocardial ischemia and reperfusion (MI/R) [38]. In the present study, there was a significant decrease (*p* < 0.01) in plasma NO, NOS and increase in plasma IL-1, IL-8 and IL-10 levels in ISO control rats (group II) as compared to group I. Quercetin (50, 100 and 150 mg/kg b.w.) pretreatment significantly increase (*p* < 0.01) plasma NO, NOS, IL-10 level and decrease plasma IL-1, IL-8 levels in group ISO + quercetin.

Quercetin is reported to possess potential antioxidant properties. The cardioprotective activity of quercetin has been attributed largely to the antioxidant properties, which are known to augment GSH and antioxidant enzyme levels and scavenge lipid peroxides. The quercetin was expected to have marked myocardial protective activity. A concept is now emerging of “adaptogenic drugs”-drugs that increases non-specific resistance to variety of stresses. Plants like *Withania somnifera* and *Bacopa monniera* show cardioprotective effects via adaptation which included augmentation of antioxidant enzymes (SOD, CAT& GSHPx), endogenous antioxidants (GSH) and stress proteins (HSP72) in the heart [39,40,41,42] Therefore, myocardial adaptation may be a promising approach to reduce cellular injury due to myocardial infarction [43]. 

The effects of ISO on heart are mediated through h1- and h2-adrenoceptors. Both h1- and h2-adrenoceptors mediate the positive inotropic and chronotropic effects to h adrenoceptor agonists [44]. Thus, ISO produces relative ischemia or hypoxia due to myocardial hyperactivity and coronary hypotension [45], and induce myocardial ischemia due to cytosolic Ca^2+^ overload [46]. Additionally, ISO causes myocardial ischemia due to excessive production of free radicals resulting from oxidative metabolism of catecholamines [47]. Grimm *et al*. [48] have reported that a toxic dosage of ISO caused characteristic myocardial damage that subsequently resulted in heart failure. In the present study, chronic administration of quercetin resulted in a increase in myocardium GSH level, SOD, GSH-Px & CAT activity and a decrease in myocardium MDA level with 50, 100 and 150 mg/kg doses. This adaptogenic property may be contributing to its cardioprotective effect and strengthen the defense mechanisms of the heart. Quercetin demonstrated cardioprotective activity, in the present study, which may be due to its direct free radical scavenging activity.

## 4. Experimental

### 4.1. Material

Quercetin was purchased from the Nanjing ZeLang Medicine Science Technology Ltd. (Nanjing, China)

### 4.2. Experimental Procedure

Wistar rats after acclimatization (6–7 days) in the animal quarters were randomly divided into five groups of eight animals each and treated as follows:

Group I—termed as normal control, received distilled water (0.5 mL/kg, b.w.) on the 29th and 30th days at an interval of 24 h.

Group II—termed as ISO control, received two injections of ISO (70 mg/kg) on the 29th and 30th days at an interval of 24 h.

Group III, IV, V—termed as ISO + quercetin, received quercetin (50, 100, 150 mg/kg, b.w.) daily for 30 days and in addition received ISO (70 mg/kg) on the 29th and 30th days at an interval of 24 h.

During the experimental period, all rats were housed under standard conditions (temperature 20 ± 1 °C, humidity 60 ± 10%, light from 6 a.m. to 6 p.m.) and given standard rodent chow and free access to water. Rats were weighed and put down 24 h after the final subcutaneous injection of ISO. Blood was collected by cardiac puncture under light ether anesthesia and allowed to clot for 30 min at room temperature. The serum was separated by centrifugation at 2,500 rpm at 30 °C for 15 min and used for the estimation of aspartate aminotransferase (AST), lactate dehydrogenase (LDH), Creatine kinase (CK-MB), NO, NOS, TNF-α, IL-1, IL-8 and IL-10.

The hearts were dissected out immediately, chilled, and perfused with ice-cold saline. After washing with ice-cold saline, the hearts were patted dry, weighed. Heart was used to prepare 10-percent (w/v) homogenate in phosphate buffer (50 mM, pH 7.4). The homogenates were centrifuged at 7,000 × g for 10 min at 4 °C and the supernatants were used for the assays of MDA, GSH, Na^+^-K^+^-ATPase, Ca^2+^-Mg^2+^-ATPase, MPO, superoxide dismutase (SOD), catalase (CAT), and glutathione peroxide (GPx).

### 4.3. Biochemical Parameters

AST, CKMB, LDH, TNF-α, NO, NOS, IL-1, IL-8, IL-10 were measured with commercially available kits which were purchased from NanJing JianCheng Bioengineering Company (NanJing, China). The assay was performed according to the manufacturer’s instructions

Na^+^-K^+^-ATPase and Ca^2+^-Mg^2+^-ATPase activities were assayed spectrophotometrically by measuring the amount of inorganic phosphate liberated following incubation of the tissue extract with disodium ATP (Sigma, Bristol, UK) as in previous studies [49]. 

Lipid peroxides were measured by the TBARS method [50]. The results were expressed in nmol MDA per mL of nmol MDA per g of tissue.

The activity of SOD was assayed as described by Kakkar *et al*. [51]. A unit of the enzyme activity was defined as the enzyme reaction giving 50% inhibition of NBT reduction in 1 min under the assay conditions and expressed as specific activity in units/mg protein.

Reduced GSH was assayed by the method of Jollow *et al*. [52,53]. An aliquot of 1.0 mL of heart PMS (10% w/v) was precipitated with 1.0 mL of sulphosalicylic acid (4% w/v). The samples were kept at 4 °C for 1 h and then centrifuged at 1,200 g for 15 min at 4 °C. The assay mixture contained 0.1 mL filtered aliquot, 2.7 mL phosphate buffer (0.1 M, pH 7.4) and 0.2 mL DTNB (40 mg/10 mL of phosphate buffer 0.1 M, pH 7.4) in a total volume of 3.0 mL. The yellow color developed was read at 412 nm.

The catalase activity was estimated by the procedure of Sinha [54], based on the fact that dichromate in acetic acid is reduced to chromic acetate when heated in the presence of hydrogen peroxide with the formation of perchromic acid as an unstable intermediate. The chromic acetate thus produced is measured at 570–610 nm.

Myocardial myeloperoxidase (MPO) activity was measured according to the method previously described [55,56]. The tissue was homogenized in Tris buffer containing proteinase inhibitors (see below) and homogenates were centrifuged for 30 min at 4,000 g at 4 °C. An aliquot (20 mL) of the supernatant was then allowed to react with a solution of tetramethylbenzidine (1.6 mM) and 0.1 mM H_2_O_2_. The rate of change in absorbance was measured with a spectrophotometer at 620 nm.

GSH-Px activity was determined with a GSH-Px Assay Kit A005 (Institute of Biological Engineering of Nanjing Jianchen, Nanjing, China). The GSH-Px had the ability to decompose hydrogen peroxide (H_2_O_2_) and other organic hydroperoxides (ROOH). The reaction uses glutathione to complete the reaction. Hydrogen peroxide, or H_2_O_2_, was used as substrate of glutathione. Consumption of nicotinamide adenine dinucleotide phosphate (NADPH) was used to determine the GSH-Px activity.

### 4.4. Histopathological Studies

At the end of the experiment, myocardial tissue was immediately fixed in 10% buffered formalin solution. The sections obtained were stained with hematoxylin and eosin (H&E) and visualized under light microscope to study the light microscopic architecture of the myocardium. 

### 4.5. Statistical Analysis

All values were expressed as mean ± S.E.M. Statistical analysis was performed using SPSS software (version 13.0.1; SPSS Inc, Chicago, IL, USA). A p value less than 0.05 was considered statistically significant.

## 5. Conclusions

Quercetin demonstrated strong cardioprotective activity by enhancing antioxidant enzymes activities and stimulating immunity function in ISO rats.
